# Partial sternotomy for management of iatrogenic brachiocephalic trunk injury during tracheotomy

**DOI:** 10.1590/1677-5449.008517

**Published:** 2018

**Authors:** Monna Hessen Banna de Oliveira, Bianca Damasceno Gonçalves, Adenauer Marinho de Oliveira Góes

**Affiliations:** 1 Centro Universitário do Estado do Pará – CESUPA, Belém, PA, Brasil.

**Keywords:** sternotomy, vascular system injury, lesions, brachiocephalic trunk, tracheotomy

## Abstract

The median thoracotomy is an access incision made longitudinally through the sternum and variants can be subdivided into total vertical and partial upper or partial lower vertical incisions. In surgical practice, using a partial median sternotomy is an alternative option that causes less surgical aggression. The brachiocephalic artery is one of the thoracic vessels most often affected in traumas and it can be accessed via a median sternotomy. This report describes use of an upper partial sternotomy to provide access in a case of traumatic iatrogenic injury of the brachiocephalic trunk.

## INTRODUCTION

 A longitudinal sternotomy may be total, extending from the sternal notch to the xiphoid process; partial upper sternotomy, in an inverted T shape over the manubrium; partial lower, with a T-shaped incision over the sternum body. 1 Median sternotomy is common in cardiovascular operations and became one of the most often performed surgical procedures worldwide with the advent of myocardial revascularization in 1967. 2

 In surgical practice, a partial median sternotomy is an alternative option involving less surgical aggression and, in selected cases, may be sufficient for the degree of exposure planned by the surgeon. Furthermore, it offers favorable esthetic results and reduced length of hospital stay. 2

 The major thoracic vessels are the thoracic segments of the aorta and the superior and inferior vena cavae, the brachiocephalic arterial trunk, the venous brachiocephalic trunks, the pulmonary veins and arteries, and the azygos vein. 3 Injuries to the major thoracic vessels account for 10% of all vascular traumas and 80% of these patients die at the scene of the traumatic event, while mortality among those who survive to hospital exceeds 20%. More than 80% of injuries to the major thoracic vessels are caused by penetrating traumas; the resulting hemorrhage may be external bleeding, bleeding contained by hematoma and mediastinal structures, cardiac tamponade, or via fistulas into adjacent structures. 3

 Hemorrhages, pneumothorax, and injuries to the trachea wall are among the most common fatal complications of tracheotomy. While venous bleeding is more common, arterial injuries with massive bleeding can also occur. 4 The brachiocephalic arterial trunk is the largest caliber branch of the aortic arch; its location and contact with adjacent structures makes it susceptible to trauma in many different situations. Although a rare complication, iatrogenic injury to this vessel can occur during tracheotomy. 4 ^-^ 6 The brachiocephalic trunk is one of the major thoracic vessels that is most often damaged by trauma. The classical approach is via a median sternotomy, which can be extended to cervicotomy. 3

 This study describes an alternative, less invasive, access option for use in cases of traumatic injury to the brachiocephalic trunk. 

## PART I – CLINICAL SITUATION

 The patient was a young male, who had been on prolonged oral endotracheal intubation after polytrauma with traumatic brain injury. The general surgery team attempted to perform a bedside tracheotomy in the intensive care unit (ICU). The procedure was begun with a longitudinal median cervicotomy, but was abandoned because of bleeding in spurts of bright red blood, and the patient was taken to the operating suite. 

 In the operating room, the cervicotomy was extended, following the anterior margin of the right sternocleidomastoid, because the diagnostic hypothesis was iatrogenic injury to the right common carotid, and vascular surgery support was requested. The vascular surgery team found that there was no damage to the right carotid and that the bleeding originated from a puncture injury to the anterior wall of the brachiocephalic trunk, partially controlled by digital compression and by placement of dressings via the cervicotomy. However, there was insufficient exposure to achieve proximal control and safely perform arteriorrhaphy. 

## PART II – WHAT WAS DONE

**Figure 1:**
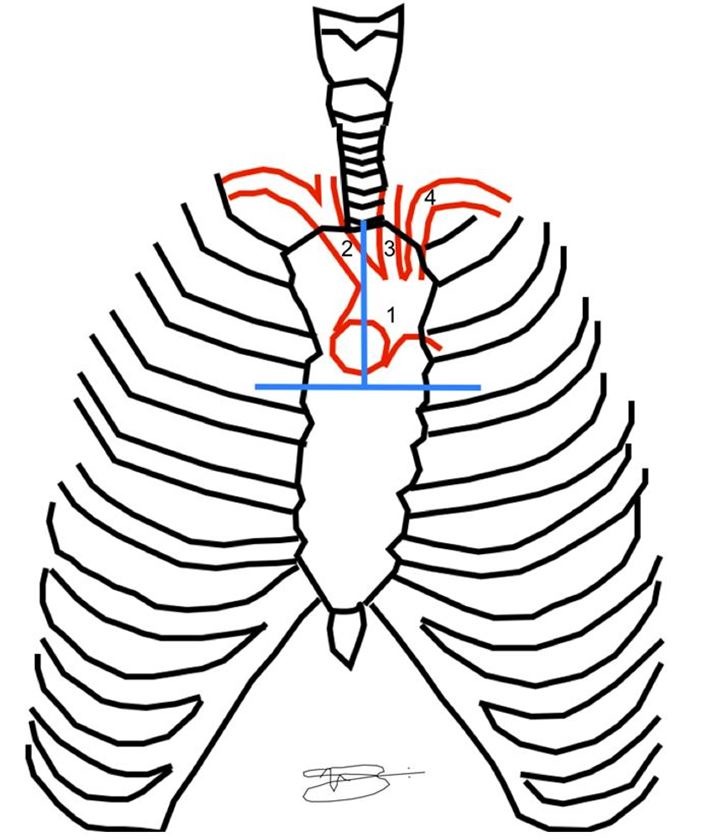
The blue lines represent the positions of bone separation for the inverted T sternotomy. 1) Aortic arch, 2) Brachiocephalic trunk, 3) Left common carotid artery, 4) Left subclavian artery.

 The decision was taken to extend the access by sternotomy. An incision was made along and the mid-sternal line from the sternal notch to the level of the third intercostal space and opened by anatomic layers. In the intercostal space, a Gigli saw was positioned transversely across the sternum with the aid of Mixter forceps and a transverse sternotomy was performed. The Mixter forceps were then passed behind the sternum, from the transverse sternotomy to the sternal notch, where the Gigli saw was positioned to perform a longitudinal sternotomy, resulting in an inverted T sternotomy ( Figure 1 ). A Finochietto retractor was placed between the longitudinal sternotomy edges and opened. 

**Figure 2:**
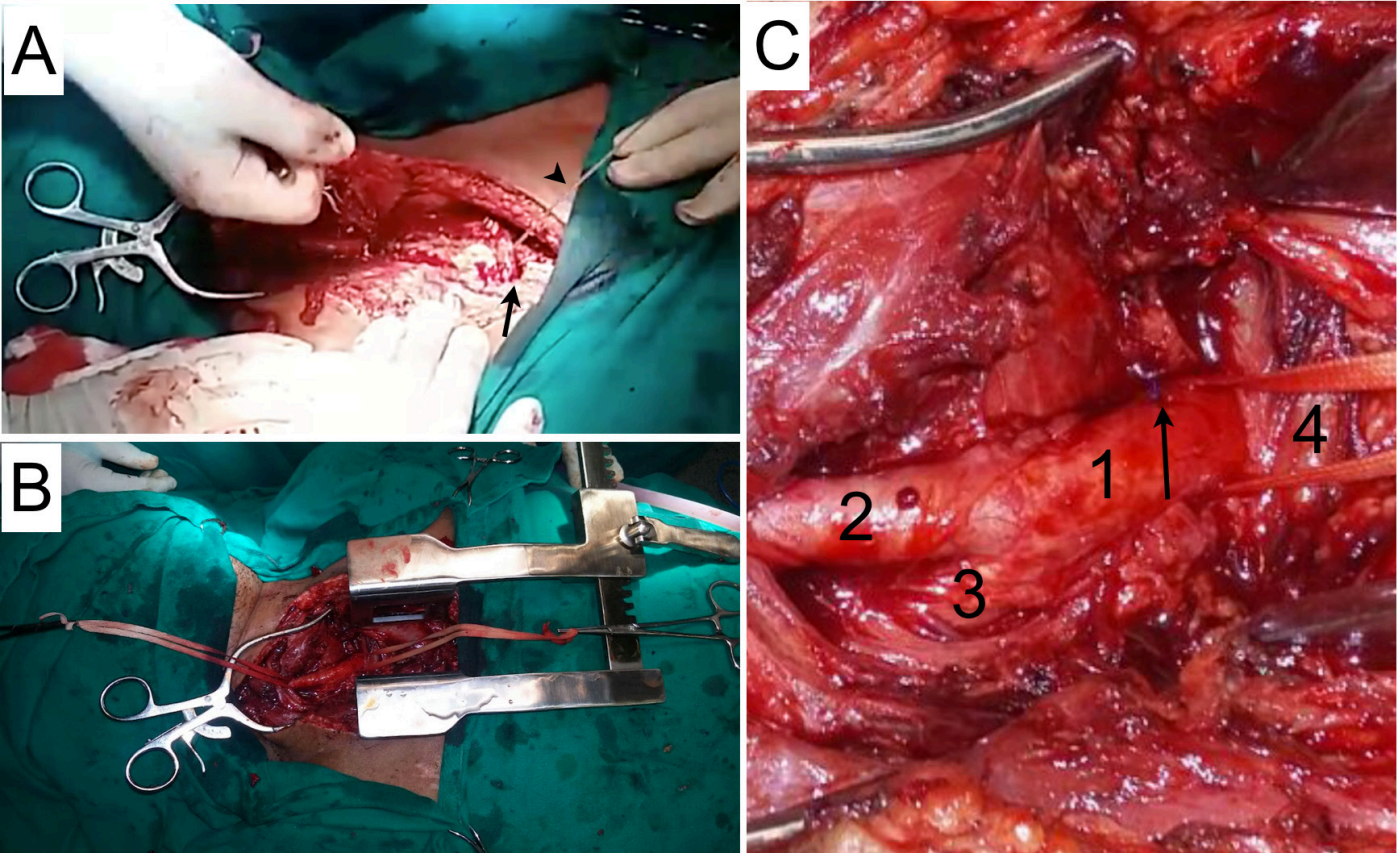
(A) Longitudinal sternotomy: the arrowhead indicates the Gigli saw and the arrow indicates the transverse sternotomy performed earlier; (B) Panoramic view of the operating field; (C) Structures in the operating field: 1) Brachiocephalic trunk, 2) Right common carotid artery, 3) Left subclavian artery, 4) Left brachiocephalic vein. Sutured lesion arrowed.

 This access provided adequate exposure of the superior mediastinum ( Figure 2 ). The brachiocephalic trunk bleeding was controlled proximal to the injury by arteriorrhaphy with one polypropylene 3.0 suture in X, achieving hemostasis. The sternum was closed with five steel wire sutures (one suture on each side of the transverse sternotomy and three along the longitudinal sternotomy). The tracheotomy was completed and the access was closed by anatomic layers. Mediastinum drainage was not performed. The patient died from systemic complications five days later. 

## DISCUSSION

 Elective bedside tracheotomy in the ICU is a safe procedure, with rates of perioperative complications of around 3%. 7 Factors that increase the risk of severe hemorrhage include percutaneous tracheotomies conducted without monitoring by bronchoscopy, low incisions, coagulopathies, and prior surgical procedures and radiotherapy involving the neck. 4

 During the tracheotomy, the surgeon should bear in mind the possible anatomic variations of adjacent vessels (brachiocephalic vessels, subclavian vessels, common carotids, and jugulars) that can increase the risk of iatrogeny. 4 ^,^ 5 While hemorrhage caused by injuries to the brachiocephalic trunk is rare during tracheotomy, 4 this artery is the second most frequently injured of the major thoracic vessels when non-iatrogenic traumas are analyzed, occurring in approximately 9% of penetrating wounds to the thorax. Among venous injuries, the left brachiocephalic vein is involved three times more often than the right, because of its greater size and its position crossing the upper mediastinum. In terms of symptomology, patients may have neurological deficits and severe hypotension or, more rarely, they may be asymptomatic. 3

 The traditional access route to these vessels is total median sternotomy with expansion to a right anterior cervicotomy (primarily for arterial injuries). Venous injuries can be repaired or ligated without major repercussions and, although it was not necessary in the case described here, tactical ligature of the left brachiocephalic vein may be necessary to improve exposure of underlying structures. 3

 In the case of iatrogenic injuries during tracheotomy, it is common for the surgeon to become aware of an injury because of unusually voluminous bleeding. In such cases, the best course of action is to apply compression, halt the bedside procedure and transport the patient to the operating room, where the situation can be better controlled. Persevering with dissection in the adverse conditions of the ICU or without adequate proximal control can result in increased arterial injury. 

 The preferred method of arterial repair depends on the extent and the mechanism of injury. Large injuries may require prosthetic grafts. For less complex injuries, the decision may be taken to use a vascular patch or perform lateral arteriorrhaphy. Mortality rates among patients subjected to surgery can reach 10% and in cases of arterial ligature the rate of neurological damage is around 25%. 3

 Sternotomy was introduced for cardiovascular surgery in 1957, as an alternative to bilateral anterior thoracotomy. At the time, it offered many benefits, since it reduced the length of surgery while maintaining excellent exposure of the heart and also reduced respiratory complications. 2 However, despite the advantages, over the years many postoperative complications have been described in relation to median sternotomy. Of these, the most significant is the risk of dehiscence of the sternal synthesis and infection (superficial, osteomyelitis, or mediastinitis). 8

 In the case described here, the choice of partial upper sternotomy (inverted T-shaped) was intended to reduce the area exposed. It was hoped that this approach would reduce thoracic cavity contact with the external environment and therefore the risk of infection. Additionally, since a shorter segment of the sternum is sawn, synthesis is achieved more quickly and dehiscence is less likely. 8 A longer than necessary incision in the sternum also increases the risk of iatrogenic injuries. Whenever possible, the sternotomy should be performed with an electric saw, but when unavailable in emergency situations, the Gigli saw is used instead. 

 The minimally invasive sternotomy described by Mulinari et al. 2 in 1997 has been considered a safe alternative access for several types of heart operations. More recently, some studies have suggested that it reduces mortality in specific groups of heart patients, including patients undergoing mitral reoperation, obese patients, and elderly patients undergoing aortic procedures. 9 Adapting what has been described for elective heart surgery, this case presents use of a safe access in a vascular trauma. 

## FINAL COMMENTS

 Surgeons performing tracheotomies should be prepared to quickly recognize a iatrogenic vascular injury and intervene to correct it. The access proposed is a viable and less invasive option for treating injuries to structures of the superior mediastinum. It can be employed in heart surgery, as previously described in the literature, but also in selected cases of vascular trauma and injuries to structures in this area. 
