# Supplementary material for: Partial sternotomy for management of iatrogenic brachiocephalic trunk injury during tracheotomy
Source: J Vasc Bras. 2018 Apr-Jun;17(2):148–51. doi: 10.1590/1677-5449.008517 (PMC6205711; doi:10.1590/1677-5449.008517)
Supplement: Translation

# Translation: Esternotomia parcial para tratamento de lesão iatrogênica do tronco braquiocefálico durante traqueostomia

**Authors:** Monna Hessen Banna de Oliveira, Bianca Damasceno Gonçalves, Adenauer Marinho de Oliveira Góes

**Affiliations:** 1 Centro Universitário do Estado do Pará – CESUPA, Belém, PA, Brasil.

**Keywords:** esternotomia, lesões do sistema vascular, lesões, tronco braquiocefálico, traqueostomia

## Abstract

A toracotomia mediana é uma via de acesso que atravessa longitudinalmente o esterno e pode ser subdividida em vertical total, parcial superior e parcial inferior. Na prática cirúrgica, o uso da esternotomia mediana parcial é uma alternativa que proporciona menor agressão cirúrgica. O tronco braquiocefálico é um dos grandes vasos torácicos mais acometidos em traumas e sua abordagem classicamente é feita por esternotomia mediana. Neste trabalho, apresenta-se o uso da esternotomia parcial superior em “T” invertido como possibilidade de via de acesso em situação de lesão traumática iatrogênica do tronco braquiocefálico.

## INTRODUÇÃO

 A esternotomia longitudinal pode ser do tipo total, que se estende desde a fúrcula até o apêndice xifoide; do tipo parcial superior, em forma de T invertido sobre o manúbrio; e do tipo parcial inferior, com incisão em T sobre o corpo do esterno 1 . A esternotomia mediana é comum em operações cardiovasculares e tornou-se um dos procedimentos cirúrgicos mais realizados em todo o mundo com o advento da revascularização miocárdica em 1967 2 . 

 Na prática cirúrgica, o uso da esternotomia mediana parcial é uma alternativa que proporciona menor agressão cirúrgica e que, em casos selecionados, pode ser suficiente para a exposição pretendida pelo cirurgião. Além disso, possibilita um resultado estético favorável e diminui o tempo de internação 2 . 

 Entende-se por grandes vasos torácicos os segmentos torácicos da aorta e cavas superior e inferior, tronco arterial braquiocefálico, troncos venosos braquiocefálicos, veias e artérias pulmonares e veia ázigos 3 . Lesões dos grandes vasos torácicos representam 10% de todos os traumas vasculares, sendo que 80% dos doentes morrem no evento traumático e os sobreviventes que chegam ao hospital têm uma taxa de mortalidade acima de 20%. Mais de 80% das lesões dos grandes vasos torácicos são causadas por trauma penetrante; a hemorragia resultante pode se apresentar de forma exanguinante, contida por um hematoma e estruturas mediastinais, tamponamento cardíaco ou ainda na forma de fístula para estruturas adjacentes 3 . 

 A hemorragia, o pneumotórax e a lesão da parede da traqueia estão entre as complicações fatais mais comuns da traqueostomia. Embora o sangramento venoso seja mais comum, lesões arteriais com sangramento profuso podem ocorrer 4 . O tronco arterial braquiocefálico (artéria inominada) é o ramo mais calibroso do arco aórtico; sua localização e contato com estruturas adjacentes a torna suscetível ao trauma em diversas situações. Embora seja uma complicação rara, a lesão iatrogênica desse vaso pode ocorrer durante a realização de uma traqueostomia 4 ^-^ 6 . O tronco braquiocefálico é um dos grandes vasos torácicos mais acometidos por trauma. A abordagem clássica da lesão é por esternotomia mediana, podendo estender-se para cervicotomia 3 . 

 Neste estudo, uma alternativa de via de acesso menos invasiva em situações de lesão traumática do tronco braquiocefálico é apresentada. 

## PARTE I – SITUAÇÃO CLÍNICA

 Paciente jovem, sexo masculino, com intubação orotraqueal prolongada após politrauma com traumatismo cranioencefálico associado. Foi submetido, à beira do leito na unidade de terapia intensiva (UTI), a tentativa de traqueostomia pela equipe de cirurgia geral. O procedimento teve início com uma cervicotomia longitudinal mediana, mas foi interrompido devido a sangramento em jato de coloração vermelha rutilante, e o paciente foi levado ao centro cirúrgico. 

 Na sala de operações, a cervicotomia foi ampliada acompanhando a borda anterior do esternocleidomastóideo direito devido a hipótese diagnóstica de lesão iatrogênica da carótida comum direita, e foi solicitado apoio da cirurgia vascular. A equipe de cirurgia vascular constatou que não havia lesão da carótida direita e que o sangramento era proveniente de lesão puntiforme na parede anterior do tronco braquiocefálico, parcialmente controlado pela compressão digital e pela colocação de gazes através da cervicotomia. No entanto, a exposição não era suficiente para o controle proximal e realização da arteriorrafia com segurança. 

## PARTE II – O QUE FOI FEITO

 Optou-se pela extensão da via de acesso por esternotomia. Realizou-se incisão e abertura por planos sobre a linha medioesternal desde a fúrcula até a altura do terceiro espaço intercostal. No espaço intercostal, uma serra de Gigli foi atravessada transversalmente sob o esterno, com auxílio de uma pinça Mixter, e uma esternotomia transversa foi realizada. Em seguida, a pinça Mixter foi passada em posição retroesternal desde a esternotomia transversa até a fúrcula, a partir de onde a serra de Gigli foi novamente posicionada, desta vez para esternotomia em orientação longitudinal, resultando assim em uma esternotomia em “T” invertido ( Figura 1 ). O afastador Finochietto foi posicionado e aberto entre as bordas longitudinais da esternotomia. 

**Figura 1:** As linhas azuis representam o sentido da diérese óssea para a esternotomia em “T” invertido. 1) Arco aórtico, 2) Tronco braquiocefálico, 3) Artéria carótida comum esquerda, 4) Artéria subclávia esquerda.

 A via de acesso permitiu ampla exposição do mediastino superior ( Figura 2 ). O controle do sangramento do tronco braquiocefálico na região proximal à lesão foi realizado, sendo tratado por arteriorrafia com um ponto em “X” com fio de polipropileno 3.0, obtendo-se hemostasia. A síntese do esterno foi realizada com cinco pontos de fios de aço (um ponto de cada lado da esternotomia transversal e três pontos ao longo da esternotomia longitudinal). A traqueostomia foi concluída e procedeu-se à síntese por planos anatômicos. Não foi realizada drenagem do mediastino. O paciente faleceu por complicações sistêmicas no quinto dia de pós-operatório. 

**Figura 2:** (A) Esternotomia longitudinal: a ponta da seta aponta a serra de Gigli, e a seta aponta a esternotomia transversa previamente realizada; (B) Visão panorâmica do campo operatório; (C) Estruturas no campo operatório: 1) Tronco braquiocefálico, 2) Artéria carótida comum direita, 3) Artéria subclávia esquerda, 4) Veia braquiocefálica esquerda. A seta aponta a lesão suturada.

## DISCUSSÃO

 A realização de traqueostomias eletivas à beira do leito em UTI é um procedimento seguro, com taxas de complicações perioperatórias ao redor de 3% 7 . Entre os fatores que aumentam o risco de hemorragia grave, encontram-se a realização de traqueostomias percutâneas sem acompanhamento por broncoscopia, incisão muito baixa, coagulopatias e procedimentos cirúrgicos e radioterapia prévios no pescoço 4 . 

 Durante a realização da traqueostomia, o médico deve ter em mente possíveis variações anatômicas dos vasos adjacentes (vasos braquiocefálicos, vasos subclávios, carótidas comuns e jugulares) que podem aumentar o risco de iatrogenias 4 ^,^ 5 . Embora hemorragias por lesões do tronco braquiocefálico sejam raras durante a traqueostomia 4 , essa lesão arterial é a segunda mais frequente entre os grandes vasos torácicos quando se analisam os traumas não iatrogênicos, ocorrendo em aproximadamente 9% dos ferimentos penetrantes do tórax. No que tange a lesões venosas, a veia braquiocefálica esquerda está envolvida três vezes mais que a direita, devido a seu maior tamanho e sua posição cruzando o mediastino superior. Quanto à sintomatologia, os doentes podem apresentar déficits neurológicos e hipotensão severa ou, mais raramente, estar assintomáticos 3 . 

 A via de acesso tradicional para abordagem desses vasos é a esternotomia mediana total com prolongamento para uma cervicotomia anterior direita (principalmente para lesão arterial). As lesões venosas podem ser reparadas ou ligadas sem maiores repercussões; inclusive, embora não tenha sido necessário no caso aqui apresentado, a ligadura tática da veia braquiocefálica esquerda pode ser necessária para ampliar a exposição de estruturas subjacentes 3 . 

 No caso das lesões iatrogênicas durante a traqueostomia, é frequente que o cirurgião perceba a lesão por um sangramento anormalmente volumoso. Nesse caso, o melhor a fazer é comprimir, interromper o procedimento à beira do leito e remover o paciente para o centro cirúrgico, onde a situação pode ser melhor controlada. A insistência em dissecar em condições adversas na UTI ou sem o controle proximal adequado pode aumentar a lesão arterial. 

 O reparo arterial preferencial depende da extensão e do mecanismo da lesão. Lesões extensas podem requerer enxertos com prótese. Em lesões com menor complexidade, pode-se optar por remendo vascular (*patch*) ou arteriorrafia lateral. As taxas de mortalidade nos doentes submetidos a cirurgia podem chegar a 10% e, na eventualidade de ligadura arterial, o índice de déficit neurológico é em torno de 25% 3 . 

 A esternotomia foi um acesso introduzido na cirurgia cardiovascular em 1957, como alternativa à toracotomia anterior bilateral. Na época, mostrou muitos benefícios, pois reduziu o tempo de cirurgia mantendo uma excelente exposição do coração e ainda diminuindo complicações respiratórias 2 . Apesar das vantagens apresentadas, ao longo dos anos foram descritas diversas complicações pós-operatórias da esternotomia mediana. Entre elas, o risco de deiscência da síntese esternal e infecção (superficial, osteomielite ou mediastinite) é a de maior relevância 8 . 

 No caso apresentado, a opção pela esternotomia parcial superior (em “T” invertido) teve como intuito diminuir a área de exposição. Com essa conduta, buscou-se diminuir o contato da cavidade torácica com o ambiente externo e o risco de infecção. Além disso, como um segmento menos extenso do esterno é serrado, a síntese é mais rápida e a deiscência é menos provável 8 . Uma incisão do esterno em extensão maior do que a necessária também promove o risco de lesões iatrogênicas. Sempre que possível, a esternotomia deve ser feita com serra elétrica; porém, em situações de emergência em que esse instrumento não esteja disponível, a serra de Gigli é usada como alternativa. 

 Descrita por Mulinari et al. 2 em 1997, a esternotomia minimamente invasiva era considerada uma alternativa segura de via de acesso em diversos tipos de operações cardíacas. Mais recentemente, alguns estudos sugeriram diminuição da mortalidade em grupos específicos de pacientes cardiopatas, incluindo pacientes submetidos a reoperações mitrais, pacientes obesos e pacientes idosos submetidos a procedimentos aórticos 9 . Adaptando o que já havia sido descrito para a cirurgia cardíaca eletiva, o caso apresenta a utilização de uma via de acesso segura em uma situação de trauma vascular. 

## CONSIDERAÇÕES FINAIS

 O médico que realiza a traqueostomia deve estar preparado para reconhecer precocemente uma lesão vascular iatrogênica e intervir para a sua correção. A via de acesso proposta é uma opção viável e menos invasiva para abordagem de lesões de estruturas do mediastino superior, a qual pode ser aplicada não somente em cirurgias cardíacas, como descrito anteriormente na literatura, mas também em casos selecionados de traumas vasculares e lesões de estruturas dessa topografia.
